# Mental health care in post-genocide Rwanda: evaluation of a program specializing in posttraumatic stress disorder and substance abuse

**DOI:** 10.1017/gmh.2016.12

**Published:** 2016-05-19

**Authors:** L. C. Ng, B. Harerimana

**Affiliations:** 1 The Chester M. Pierce, MD Division of Global Psychiatry, Department of Psychiatry, Massachusetts General Hospital, Boston, MA, USA; 2 Department of Psychiatry, Harvard Medical School, Boston, MA, USA; 3 Neuropsychiatric Hospital CARAES Ndera, Kigali, Rwanda

**Keywords:** Genocide, mental health services, mental illness, policy and systems, program evaluation, Rwanda

## Abstract

**Background.:**

Following the genocide, millions of Rwandans are likely living with posttraumatic stress disorder (PTSD). Le Centre Psychothérapeutique Icyizere provides the only specialized treatment for PTSD in the Rwandan healthcare system.

**Methods.:**

Demographics, diagnosis, treatment, outcomes, and scores on assessments of functioning and PTSD were recorded from clinical charts of all patients receiving care between October 2013 and 2014. Descriptive statistics and within-group *t* tests comparing functional impairment and PTSD symptoms at intake to discharge and follow-up were calculated.

**Results.:**

A total of 719 patients (55.08% male) received care. Patients were more educated, more likely to live in the capital, and less likely to be married than the general population. Patients reported high desire for, and strong satisfaction with, care. Most patients (55.60%) were still in care by the end of the program evaluation. Functioning improved from intake to discharge (*p* < 0.001), and improvements were sustained at follow-up (*p* < 0.001). Most adults were diagnosed with psychotic disorders, substance use disorders, or depression. Only 20 patients were diagnosed with PTSD, and symptoms were improved at discharge (*p* = 0.003).

**Conclusions.:**

This program evaluation demonstrated the utility of a low-resource information management system to provide clarity about the patient population and outcomes. Results suggest that services are effective and sustainable, although people with PTSD were not the primary recipients of care. Disparities in care by diagnosis, education, marital status, and geography are discussed. Results have contributed to changes in service delivery and care and efforts to increase access to care.

Mental health in Rwanda has been deeply impacted by the 1994 Rwandan Genocide, in which approximately one million people were killed, 250 000 women were raped, and millions of Rwandans were displaced (Pham *et al.* 2004; Republic of Rwanda, 2012; Binagwaho *et al.* 2014). Survivors were exposed to extreme levels of physical and psychological violence (Ng *et al.*, 2015). Studies have reported that 94% of people in Rwanda during the genocide experienced at least one genocide event including witnessing the murder of family members, having their property and homes destroyed, and having their lives threatened (Pham *et al.* 2004). As a result of these experiences, traumatic stress symptoms related to the genocide are highly prevalent. Approximately 25% of the population is estimated to meet criteria for posttraumatic stress disorder (PTSD) (Pham *et al.* 2004).

Over the last 10 years, the Rwandan government has invested heavily in delivery of quality health care, and physical health indicators have improved dramatically (Saksena *et al.* 2011; Drobac *et al.* 2013; Binagwaho *et al.* 2014). However, like most low-income countries (Kebede *et al.* 2003; Wang *et al.* 2007; Eaton *et al.* 2011), Rwanda had almost no mental health treatment services prior to, or following, the genocide (World Health Organization. Dept. of Mental Health Substance Abuse, 2005). The availability of mental health services is still very limited due in part to a lack of trained professionals and a small budget for mental health services (World Health Organization. Dept. of Mental Health Substance Abuse, 2005; Department of Mental Health and Substance Abuse, WHO, 2011; Umubyeyi *et al.* 2016).

The Rwandan mental health care system consists of two agencies that provide specialized mental health care: CARAES Ndera Neuro-Psychiatric Hospital, the national referral hospital for neuropsychiatric disorders, and the outpatient Psychosocial Consultation Service (Service de consultation psychosociale; SCPS) (Department of Mental Health and Substance Abuse, 2011; Republic of Rwanda Ministry of Health, 2011). Both CARAES Ndera and the SCPS primarily serve patients with epilepsy, severe mental illness, psychosomatic disorders, and neurological disorders (Republic of Rwanda Ministry of Health, 2011). In addition, the government has been actively decentralizing health care, and recently the district hospitals have started employing mental health nurses and psychologists. Mental health professionals have also been training health center staff in diagnosing and treating mental disorders, but the great majority of mental health care is still done at the national hospital level and few people are seen for care in health centers (Umubyeyi *et al.* 2016).

Although it is likely that millions of Rwandans are affected by PTSD symptoms (Pham *et al.* 2004), Rwanda did not have specialized treatment for PTSD available until 2004, 10 years after the genocide. In 2004, CARAES Ndera created a service branch called Le Centre Psychothérapeutique Icyizere (The Hope Center), whose mission is to serve patients with PTSD. In 2010, substance abuse treatment and detoxification were added to the package of care to address the needs of dual-diagnosis patients. Icyizere still provides the only specialized treatment for substance abuse and PTSD in the Rwandan health care system.

Icyizere's services include inpatient and outpatient medication management, individual, group and family therapy, and detoxification. In addition, staff are engaged in mental illness prevention through community outreach programming. The health providers at Icyizere are primarily bachelor's degree level psychiatric nurses and psychologists who receive ongoing continuing education trainings through workshops organized by the international NGO Fracarita International and other partners. In addition, Icyizere disseminates information about diagnosing and treating PTSD and substance abuse to health professionals working in district hospitals and health centers and provides supervision and practicum experiences to high school and university students in nursing and psychology.

The Rwandan government has embraced a model of evidence-based care and emphasizes the need for, and use of, routine assessment of clinical services (Republic of Rwanda, 2014; Rwandan Research and Implementation Writing Group, 2014). All Rwandan health service providers are expected to provide data to the Rwandan Ministry of Health on enrolled patients and care (Department of Mental Health and Substance Abuse, WHO, 2011; Republic of Rwanda, 2014). Routine monitoring of patients and services is critical for measuring service delivery outcomes and for guiding future policy, programming, and investment (Gonzalez Block & Mills, 2003; Chisholm *et al.* 2007; Saraceno, 2007; Minas, 2012). However, like most low-income countries (Razzouk *et al.* 2010; Minas, 2012), Rwanda has a shortage of trained clinical science researchers, and health providers have limited time and resources to invest in providing intensive research training to their staff (Rwandan Research and Implementation Writing Group, 2014). As a result, as of 2014 only 8% of health centers and district hospitals had an information management system for tracking patient data and no health center had a quality assurance team (Republic of Rwanda, 2014). Therefore, despite the mandate to regularly evaluate health programs using patient-level data, until this program evaluation, Icyizere did not have a data monitoring and evaluation program, and was therefore unable to comprehensively report on the state of PTSD care in Rwanda.

Although Rwanda has been providing treatment for PTSD for over 10 years, this is the first study to report on the clinical care provided by a PTSD and substance abuse treatment center in Rwanda. This study is a program evaluation of Icyizere using routinely collected data. The program evaluation was conducted from October 2013 to October 2014, and was Icyizere's first time identifying, gathering, summarizing, and evaluating key patient-level indicators of treatment for PTSD in the Rwandan health care system.

## Methods

### Chart review

Data corresponding to the key indicators were obtained from clinical notes and patient charts during a chart review performed by an Icyizere data officer who had previously been a psychology trainee at Icyizere. The data officer used medical records and appointment logs to identify all patients seen at any time at Icyizere between October 2013 and October 2014 and extract demographic information, diagnosis, treatment information, and clinical outcomes. If patients had been seen prior to October 2013, they were identified as returning patients however data were only culled from records of appointments during this time period. Similarly, data from follow-up appointments after October 2014 were not included in this program evaluation. The information was only used for clinical purposes and clinicians only had access to information on their own patients. All data were entered into a password-protected excel spreadsheet for analysis without patient names. The spreadsheet was located on a password-protected computer accessible only to the data officer and the clinic director.

### Key indicators

One of the primary goals of the program evaluation was to develop and implement a simple, sustainable, and integrated information system to be used in Icyizere after the conclusion of the formal program evaluation. To ensure that the system would meet Icyizere's specific monitoring and evaluation or quality improvement needs, the director identified a core set of key indicators that were tailored to the needs of their service and patient population (Gonzalez Block & Mills, 2003). These included demographic, diagnostic, and treatment indicators, described below.

#### Demographics

Patient demographic information included patient sex, age, marital status, number of children, education, home province, health insurance, and out of pocket costs for patient care.

#### Primary diagnosis

Primary psychiatric and substance abuse diagnoses were recorded. Patients with dual-diagnoses of at least one psychiatric disorder and one substance abuse disorder were categorized as dual-diagnosis.

#### Treatment

Information on treatment included whether the patient was new to Icyizere or a longer term patient, previous treatment received, referral source to Icyizere, patient's interest in, and motivation for, treatment, whether the patient was receiving medication, therapy, or both, the number of follow-up sessions, and whether a patient remained in care, was discharged or referred, or was lost to follow-up by the end of the program evaluation.

#### Functional impairment

The functional impairment of patients receiving inpatient services was assessed by clinical staff, when admitted to the inpatient unit, at discharge from the inpatient unit, and at the final outpatient follow-up visit using the 36-item WHO Disability Assessment Schedule 2.0 (WHODAS 2.0) (World Health Organization, 2012). Items are scored 1 (‘none’) to 5 (‘extreme’) and Icyizere used simple scoring, recording the average score at each assessment period in the clinical chart. The WHO-DAS was not administered to outpatient clients, and so data are unavailable on their functional impairment.

#### PTSD symptoms

Symptoms of patients diagnosed with PTSD were assessed by clinical staff at intake for new outpatients or when admitted to the inpatient unit for inpatients, at discharge from the inpatient unit for inpatients, and at the final follow-up session for both inpatients and outpatients using the PTSD Checklist-Civilian Version (PCLC) (Weathers *et al.* 1994). The 17 items on the scale were scored 1 (not at all) to 5 (extremely) and were summed for a total score of 17–85.

### Data analysis

Descriptive statistics were calculated for all of the key indicators. Within-group *t* tests were conducted to compare functional impairment and PTSD symptoms at baseline to discharge, and to compare intake to follow-up. Analyses were conducted with SPSS version 22 (IBM Corp., 2013) and Stata version 14 (StataCorp LP, 2015).

### Ethics approval

The program evaluation was part of the routine clinical services of Icyizere for the purpose of quality improvement, self-assessment, and evidence-based decision-making process and therefore did not require formal ethics board approval. The General Directorate of CARAES Ndera gave consent to the program evaluation and approval to publish the results. The director of Icyizere commissioned and oversaw the evaluation as part of his official duties. The data officer who performed the data extraction was a member of the Icyizere clinical staff. Patient medical records and clinical notes were handled to ensure confidentiality and the data extracted were recorded without patient names.

## Results

### Demographics

Between 29 October 2013 and 20 October 2014, 719 patients (55.08% male) were seen for clinical services at Icyizere (see Table 1). Patients ranged in age from 2 to 93 with an average age of 32.76 (s.d. = 15.09) and 11.96% were under age 18. Most of the adults were single (63.71%) and childless (62.38%). On average, the adults had 11.73 (s.d. = 4.54) years of education. Approximately two-thirds of adults completed secondary school (64.01%) and one-fifth (22.70%) completed university. Three-quarters of the patients (74.79%) lived in the capital city, Kigali, and only 1.54% of patients were not from Rwanda. Almost all (99.30%) of the patients had health insurance, and three-quarters (73.64%) paid <250 RWF ($0.37 USD in April 2014) for their care at Icyizere.

**Table 1.** Patient demographics

Key demographic indicator (*N* = 719)	Total, *n* (%)
Male	396 (55.08)
Age	Mean = 32.76, s.d. = 15.09
Child (0–17)	86 (11.96)
Young adult (18–24)	148 (20.58)
Adult (25–64)	458 (63.70)
Older Adult (>65)	27 (3.76)
Marital status (ages 18 and older), *n* = 631	
Single	402 (63.71)
Married	191 (30.27)
Divorced	13 (2.06)
Widowed	25 (3.96)
Number of children (ages 18 and older), *n* = 630	Mean = 1.28, s.d. = 2.12
None	393 (62.38)
1–3	141 (22.38)
4–6	72 (11.43)
>=7	24 (3.81)
Years of education (*n* = 638)	Mean = 11.01, s.d. = 4.92
None	39 (6.11)
Some primary (P1-P5)	48 (7.52)
Completed primary (P6)	36 (5.64)
Some secondary (P7/S1- S5)	149 (23.35)
Completed secondary (S6)	154 (24.14)
Some university	70 (10.97)
Completed university (A0)	121 (18.97)
Postgraduate	8 (1.25)
Province (*n* = 714)	
Kigali	534 (74.79)
North	30 (4.20)
South	66 (9.24)
East	44 (6.16)
West	29 (4.06)
Other East African countries	11 (1.54)
Health insurance (*n* = 717)	
Mutuelle	519 (72.38)
Private	65 (9.07)
RSSB	97 (13.53)
Other	31 (4.32)
None	5 (0.70)
Out of pocket cost of treatment in RWF (*n* = 717)	Mean = 1124.52, s.d. = 3297.98
0	6 (0.84)
240	522 (72.80)
250–1000	118 (16.46)
7200	68 (9.48)
>7200	3 (0.42)

### Diagnosis

**Fig. 1.**
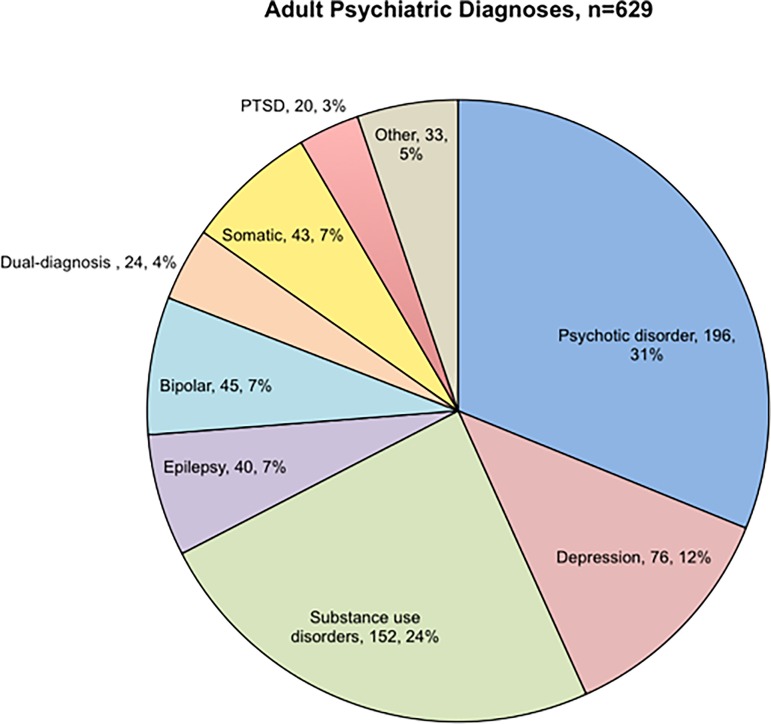
Adult diagnoses (*n*, %).

**Fig. 2.**
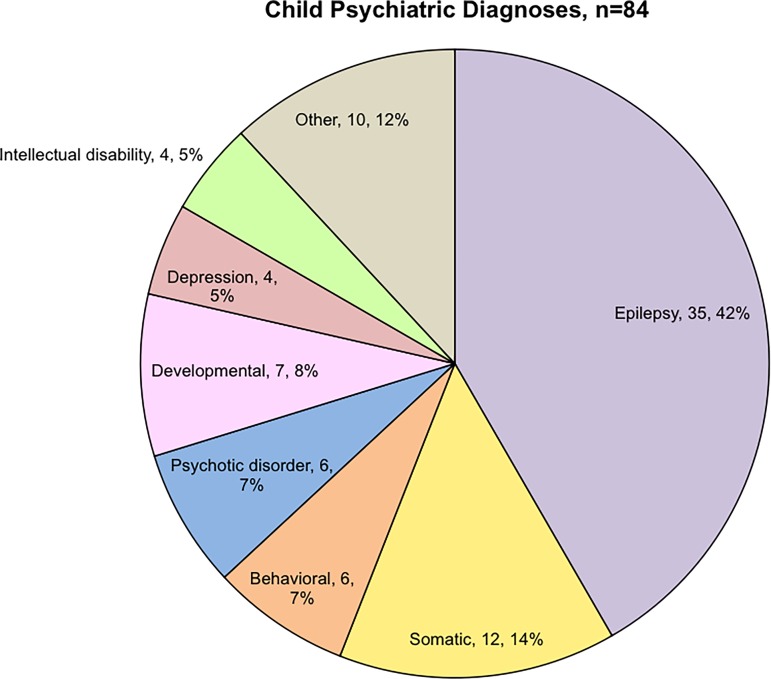
Child diagnoses (*n*, %).

The most frequent diagnoses of adult patients were psychotic disorders (31.16%), substance use disorders including substance-induced psychosis (24.17%), depression (12.08%), bipolar disorder (7.15%) and epilepsy (6.36%) (see Fig. 1). Amongst all adult patients, 12.88% abused alcohol and 6.20% abused cannabis (including 3.66% of patients who abused both). Other drugs such as heroin and cocaine were abused by 3.34% of patients. Dual-diagnoses of psychiatric disorders and comorbid substance use accounted for an additional 3.82%, with most cases involving comorbid depression and alcohol abuse. For children, the most common diagnosis was epilepsy (41.67%) followed by somatic (14.29%), developmental (8.33%), behavioral (7.14%), and psychotic (7.14%) disorders (see Fig. 2).

### Treatment

About half (52.64%) of the patients were new to Icyizere (see Table 2). Prior to receiving care at Icyizere, 51.71% of patients had received previous psychiatric treatment from Ndera, SCPS, hospitals, or health centers. Most patients heard about Icyizere from family (74.58%) and one-fifth (20.53%) were referred by a health service provider. Most patients presented for care with their families (85.63%) and the great majority of patients believed that treatment was helpful, important, and/or necessary (90.26%).

**Table 2.** Treatment information

Key treatment indicator (*N* = 719)	*n* (%)
New case (*n* = 718)	378 (52.65)
Previous medical treatment other than Icyizere (*n* = 468)	
None	220 (47.01)
Ndera	120 (25.64)
Hospital	69 (14.74)
SCPS	24 (5.13)
Health Center/clinic	29 (6.20)
Traditional healer	4 (0.85)
Referral Source (*n* = 716)	
Family	534 (74.58)
Health provider	147 (20.53)
Community member	18 (2.51)
Friend	2 (0.28)
Another patient	5 (0.70)
Religious leader	1 (0.14)
Employer	9 (1.26)
Service seekers (*n* = 717)	
Both family and patient	614 (85.63)
Family	71 (9.90)
Patient	30 (4.18)
Community members	2 (0.28)
Patient attitudes towards treatment (*n* = 688)	
Helpful/important/necessary	621 (90.26)
Not helpful, important or necessary	63 (9.16)
No insight	4 (0.58)
Suicidal ideation (*n* = 718)	27 (3.76)
Past suicide attempt (*n* = 718)	24 (3.34)
Inpatient treatment (*n* = 718)	163 (22.70)
Psychotropic medication (*n* = 662)	631 (95.32)
Adherent to medication (*n* = 605)	554 (91.57)
Psychotherapy (*n* = 719)	321 (44.65)
Type of individual psychotherapy (*n* = 300)	
Cognitive behavioral therapy	130 (43.33)
Humanistic	127 (42.33)
Motivational interviewing/relapse prevention	10 (3.33)
Supportive therapy	19 (6.33)
Completed	14 (4.67)
Type of Group therapy (*n* = 103)	
Relapse prevention	85 (82.52)
Psychoeducation	15 (14.56)
Stress management	3 (2.91)

Most patients (77.30%) received outpatient services and almost all patients (95.32%) were prescribed psychotropic medications. In addition to medication, 44.65% of patients were receiving psychotherapy, with 67.91% receiving only individual, 6.54% receiving only group, and 25.55% receiving both individual and group therapy. Most of the individual therapy provided was cognitive behavioral therapy (43.33%) or humanistic (42.33%) therapy, and the group therapy was primarily focused on relapse prevention (82.52%).

Almost all patients (99.03%, *N* = 719) completed at least one follow-up session at Icyizere, and the mean number of follow-up sessions was 4.66 (s.d. = 3.73; range 0–18). In addition, information on retention in care was available for 536 patients (74.55%). Of the 536 patients, 298 (55.60%) were still in care by the end of the program evaluation, 199 (37.13%) had missed the last documented follow-up session and were lost to follow-up at the end of the program evaluation, and 39 (7.28%) had been discharged from care due to referral or improvement.

### Functional impairment

Of the 163 inpatients, 70 had their functional impairment assessed at intake. The mean functional impairment score at intake was 2.59 (s.d. = 0.73) indicating mild to moderate levels of functional impairment. On average, patients were discharged from the inpatient unit 28.81 days (s.d. = 17.53) after intake. At discharge from the inpatient unit at Icyizere, functional impairment improved to below mild levels (Mean = 1.72, s.d. = 0.69, *n* = 49). The improvement from intake to discharge was significant [*t*(47) = 7.26, *p* < 0.001]. At the last follow-up session, which was on average 71.92 days (s.d. = 31.92) after discharge, functional impairment scores continued to be mild (Mean = 1.52, s.d. = 0.65, *n* = 25) and significantly better than scores at intake [*t*(23) = 4.52, *p* < 0.001]. There was no difference between functional impairment scores at discharge and at follow-up [*t*(23) = 0.002, *p* = 0.998]. In addition, of 150 patients who were assessed at discharge, patients, families, and therapists all reported that more than 88% of the patients were improved (83–87%) or recovered (1–7%) (see Table 3). Approximately 8% were unchanged and 2% were worse after treatment.

**Table 3.** Patient, family, and therapist report on recovery following treatment, n (%)

	Worse	Same	Better	Recovered
Patient (*n* = 149)	3 (2.01)	12 (8.05)	124 (83.22)	10 (6.71)
Family (*n* = 150)	3 (2.00)	13 (6.67)	127 (84.67)	6 (4.00)
Therapist (*n* = 150)	3 (2.00)	14 (9.33)	131 (87.33)	2 (1.33)

### PTSD

Only 20 adult patients (3.18%) were diagnosed with PTSD. Two of the patients with PTSD had a comorbid alcohol use disorder (10%). Of the patients with PTSD, 14 were females (70%) and they ranged in age from 20 to 60 with a mean age of 34.75 (s.d. = 10.73). About one-third were married (30.00%). Half had completed secondary school (52.63%) and one-fifth (21.05%) had completed university. Seven of the 20 patients with PTSD were new Icyizere patients during the time of the program evaluation, and three were old patients, but were admitted as inpatients. Since these 10 patients received comprehensive intakes during the program evaluation period, they had their symptoms assessed using the PCLC at intake. Eight of the 10 patients reported that their most distressing experience was the genocide; the other two patients reported that their most distressing experiences were ‘mistreatment’ and ‘parent's death.’

Most patients with PTSD (70%) were receiving outpatient care. The great majority (85%) of patients were prescribed medications, which were tricyclic antidepressants (*n* = 10), atypical antipsychotics (*n* = 6), benzodiazepines (*n* = 4), SSRIs (*n* = 2), typical antipsychotics (*n* = 1) and phenothiazine antipsychotics (*n* = 1). Five patients were taking more than one medication. In addition to medication, 13 of the 20 patients (65%) were receiving individual therapy, of which 11 were receiving humanistic therapy and two were receiving cognitive behavioral therapy.

Scores on the PCLC indicated that at intake, all 10 patients met DSM-IV-TR (American Psychiatric Association, 2000) criteria for PTSD and scored an average of 57.50 (s.d. = 9.24), indicating moderate severity. For the five inpatients, PTSD scores were assessed at discharge from the hospital; three (60%) met DSM-IV-TR criteria and the mean PCLC score was 30.20 (s.d. = 6.72) indicating mild severity. At the last treatment follow-up for inpatients and outpatients, five patients were interviewed, three met DSM-IV-TR criteria, and the mean score was 36.80 (s.d. = 12.83) indicating mild to moderate severity. Despite the small sample size, PCLC scores at discharge were significantly lower than at intake [*t*(4) = 6.50, *p* = 0.003] and there was a trend towards PCLC scores at follow-up being significantly less than those at intake [*t*(4) = 2.40, *p* = 0.07]. There were no differences between scores at discharge and follow-up [*t*(2) = −0.28, *p* = 0.80].

## Discussion

This study describes a sustainable model of mental health care in a low-income country and the development of an excel worksheet that provided a simple information management system tailored to the needs, resources, and research capacity of a low-resource setting. The staff used the system to evaluate their program and make changes to service delivery and care, including increased use of clinical assessment and evaluation tools, improvements in identifying comorbid conditions, increased awareness by staff and clinicians about patient symptoms and treatment effects, more effort to raise community and provider awareness of the services offered by the center and how to access them, internal dialogues about patient diagnostic profiles and the need to disseminate care beyond the capital city, and a commitment to maintain and develop the information management system.

In many high- and middle-income countries, electronic medical record (EMR) systems with the capacity to provide aggregated data are used for monitoring and evaluation and quality improvement. However, at Icyizere, as in many health services in low-income countries and other under-resourced settings, setting up an EMR system was not feasible given limited staff access to computer hardware and software, the need for a consistent electrical source and an internal network, and the need to have well-trained and salaried information technology officers to maintain and monitor the system (Gonzalez Block & Mills, 2003; Chan *et al.* 2012). However, the simple and feasible approach described here produced useful and actionable results. Notably, this approach could be used at other health facilities in Rwanda, as the Rwandan government has prioritized having data managers and officers available to all health facilities (Holvoet & Inberg, 2014). While a more formal and comprehensive information management system is ideal, this process might also be used as a starting point by other low-resource service organizations to begin to plan and organize services and integrate a service-specific information gathering and monitoring system in clinical care (Saraceno, 2007).

Results of the program evaluation indicated that patients seeking care for mental health services at Icyizere were more educated and were more likely to live in Kigali than the general population in Rwanda (National Institute of Statistics of Rwanda (NISR) [Rwanda], 2015). Individuals with less education may be less likely to seek care due to stigma, lack of knowledge about mental illness and mental healthcare, and challenges accessing care due to poverty, lack of health insurance, and other barriers (Ferrari *et al.* 2015; Rugema *et al.* 2015). This disparity in access is particularly concerning because research has found that Rwandans who were most in need of mental health care were those with the least amount of education (Ng *et al.* 2015; Umubyeyi *et al.* 2016).

The observed lack of geographic diversity may be explained by inaccessibility (Huerta Munoz & Kallestal, 2012) and lack of access to, and knowledge about, mental health services in rural areas (Rugema *et al.* 2015). Although only 11% of the Rwandan population lives in the capital Kigali (National Institute of Statistics of Rwanda, 2012), almost all mental health services are located in Kigali (World Health Organization. Dept. of Mental Health Substance Abuse, 2005; Department of Mental Health and Substance Abuse, WHO, 2011). Getting to Kigali for services at Icyizere can be expensive and time consuming for patients and their families, particularly when only 16 inpatient beds are available and the majority of patients receive outpatient services requiring multiple trips to the city.

Although efforts to increase mental health services in rural primary care clinics and district hospitals are ongoing, most people are still not accessing mental health care there (Rugema *et al.* 2015; Umubyeyi *et al.* 2016). Indeed, only 20% of patients at Icyizere had been referred for care by a health provider, and only 22% had received any mental health treatment prior to accessing care at one of Rwanda's specialized mental health hospitals. Improvements in community and health provider education about mental illness and improvements in the quality of care may be needed to increase interest in accessing care in rural areas (Rugema *et al.* 2015).

An additional disparity in care that emerged from the data was that adults receiving care at Icyizere were less likely to be married and have children than adults in the Rwandan population (National Institute of Statistics of Rwanda (NISR) [Rwanda], 2015). This result may be explained by research indicating that adults with mental illness in Rwanda are less likely to marry, perhaps due in part to high rates of stigma and community rejection (Rugema *et al.* 2015). It may also be that people with mental illness who are married or have children may be less likely to seek care. Most people with mental illness in Rwanda receive care from family or community members rather than from health providers (Umubyeyi *et al.* 2016), and so those who are married or who have children may be more likely to receive care from their immediate family than to seek professional services. Finally, having a family may be protective and may reduce the likelihood of developing mental illness, the severity of mental illness, or the functional impairment associated with mental illness (Hopper *et al.* 2007; Yu & Shim, 2009).

Almost all patients and their family members believed that care was necessary and helpful, suggesting that services were desired and acceptable. Family members were involved in accessing care for 96% of patients, indicating the critical role that family involvement plays in accessing care. Greater attention and support to access to care and delivery of care for patients who do not have family support or involvement may reduce disparities in care. This may be particularly critical for access to mental health care, as high stigma and caregiver burden can result in lack of family support and delays in accessing care (Lasalvia *et al.* 2014; Gronholm *et al.* 2015; Semrau *et al.* 2015). Indeed, family awareness of mental illness and support for treatment have been identified as critical factors in patient's access to treatment in Rwanda (Rugema *et al.* 2015).

Inability to pay for health services is a common barrier to care (Saksena *et al.* 2011; Rugema *et al.* 2015). Notably, patients who accessed care at Icyizere had low out-of-pocket costs, due in large part to almost all patients being enrolled in health insurance. The mental health care provided by Icyizere is sustainable in large part because it is government run and financed, and insurance providers in Rwanda cover mental health treatment. In Rwanda, health insurance coverage is compulsory, and almost all Rwandans are enrolled (Farmer *et al.* 2013), although keeping coverage can be challenging, particularly for poorer households (Saksena *et al.* 2011). Results from this evaluation indicated that 72% of the patients served at Icyizere were able to afford their care because they were enrolled in the government run *Mutuelle de Santé* health insurance program (Saksena *et al.* 2011; Drobac *et al.* 2013; Binagwaho *et al.* 2014), which is the primary insurance provider for most Rwandans. Lack of enrollment in *Mutuelle* or another health insurance scheme has been identified as the primary structural barrier stopping people in Rwanda from accessing mental health care (Rugema *et al.* 2015; Umubyeyi *et al.* 2016). Increased enrollment in affordable health insurance schemes would be expected to increase access to mental health care for the most vulnerable.

Patients and family members who were able to access care at Icyizere reported high desire for care, and strong satisfaction with the care they received. Moreover, patient, family, and therapist reports of improvement were mirrored by improvement in functional impairment and PTSD symptoms on assessment measures. While these results are encouraging, self-reported clinical outcomes were only collected for a small number of patients and only at discharge, so results of other patients are unknown. However, one of the positive changes that developed from the program evaluation was that the administration of Icyizere began to implement broader use of outcome assessment tools to better understand the outcomes of more of the patients.

Another unexpected result from the program evaluation was the small number of patients receiving a primary diagnosis of PTSD despite the mission of Icyizere being to serve patients with PTSD. The most prevalent diagnoses among adults were psychotic and substance use disorders. Results suggest that the number of people with PTSD in Rwanda who are receiving mental health care is extremely low. Although services are available, people are not presenting to care. Reasons for the disconnect between the potential number of patients and the actual patients being seen in care could be due to lack of community awareness about PTSD and services, or an inability to access care due to geographic, financial, or individual barriers. Additionally, in many communities including those in Rwanda, mental illness is understood almost exclusively to be psychosis (Rugema *et al.* 2015), and PTSD symptoms may not be viewed as warranting mental health care. This may be particularly true in communities where the burden of trauma exposure and PTSD is extremely high, long-term mental health outcomes are heterogeneous (Karstoft *et al.* 2013), and symptoms may seem disconnected from functioning (Sack *et al.* 1999). Further research is required to understand specific factors that may be limiting treatment seeking in this setting.

Discussions with Icyizere staff also indicated that many psychotic disorders and substance use disorders were preceded by traumatic events and PTSD symptoms and these disorders may be complications from earlier trauma reactions. Indeed there is very high comorbidity between PTSD and psychosis (Grubaugh *et al.* 2011; Bajor *et al.* 2013) and PTSD and substance abuse (Schafer & Najavits, 2007; Dass-Brailsford & Myrick, 2010; McCauley *et al.* 2012), and often PTSD is undiagnosed in these populations (Frueh *et al.* 2002; Salyers *et al.* 2004; Chessen *et al.* 2011; Chernomas & Mordoch, 2013). As a result of this finding, greater attention is being paid to comorbid and underlying diagnoses at Icyizere. Another explanation may be that traumatic events predispose people to developing psychosis (Ruby *et al.* 2014) or substance abuse (Stewart, 1996; Currier *et al.* 2014). This trajectory requires more attention, particularly in communities affected by mass trauma such as post-conflict environments. Indeed psychosis, rather than PTSD, is the primary mental illness treated in refugee camps (Kane *et al.* 2014). Neglecting the association between PTSD and psychosis may be doing a disservice to communities impacted by violence and war. Further research on the relationships between trauma exposure, PTSD, psychosis, substance abuse and functioning in war-affected communities is needed.

This study has a number of limitations. As a program evaluation, there were no control groups available to compare to clinical outcomes and outcome assessments were limited to the measures already used in clinical care and to the patients who were administered them, which were primarily inpatients. In addition, clinicians did not always assess patients and there was some loss to follow-up. As a result, outcome data were only available for a fraction of the patients. This limits the internal validity of the data, but captures the current practices of the clinic.

One of the clinical changes that occurred due to the program evaluation was the increased use of outcomes tools for all patients. In addition, because the data collection system was designed to be tailored to the needs of Icyizere, standardized questions common to other mental health services studies were not used, making comparison difficult. Finally, because program evaluations only assess one service, the results are not generalizable to other services or settings. Despite these limitations, we also believe that this study has a number of strengths. This study demonstrated the utility of a simple, low resource, information management system to provide clarity about the patient population being treated and the outcomes of the services offered. In addition, the results provide the first description of mental health services available for PTSD in Rwanda and suggest that services are effective and sustainable, although people with PTSD are not the primary recipients of care. More research is needed about reasons why people with PTSD may not be seeking or accessing care, and on relationships between PTSD, severe mental illness and substance abuse in this population. However, the results have already contributed to changes in service delivery and care. Efforts to evaluate existing sustainable models of care in low-income countries may provide insight into strategies for expanding these services to other low-resourced and underserved communities.
